# Traumatic Inferior-Anterior Hip Dislocation in a 15-Year-Old Without Fracture: A Five-Year Follow-Up and Review of the Literature

**DOI:** 10.7759/cureus.80448

**Published:** 2025-03-12

**Authors:** Panagiotis Antzoulas, Anna Konstantopoulou, Pantelis Tsoumpos, Dimitris Tatarakis, George Tagaris

**Affiliations:** 1 Orthopaedics Department, University of Patras School of Medicine, Patras, GRC; 2 Orthopaedics Department, Karamandaneion Children's Hospital, Patras, GRC

**Keywords:** dislocation, hip, inferior-anterior, pediatric, traumatic

## Abstract

Traumatic hip dislocations in the pediatric population are relatively rare but potentially devastating injuries, with anterior hip dislocations being even less common. These injuries typically result from high-energy trauma and direct impact mechanisms, most frequently due to motor vehicle accidents and, to a lesser extent, sports-related injuries. In adolescents, hip dislocations are often associated with fractures of the acetabulum or femoral head, necessitating urgent reduction under general anesthesia. Delays exceeding six hours significantly increase the risk of avascular necrosis (AVN) of the femoral head. The literature contains only a limited number of reported cases. We present a rare case of traumatic inferior-anterior hip dislocation without an associated fracture in a 15-year-old male with normal body habitus. The patient was brought to the emergency department after sustaining a direct impact to the left posterior hip following a collision with a moving vehicle. He presented with severe pain and tenderness in the left hip, a restricted range of motion, complete inability to move the left hip joint, and apparent limb shortening of approximately 5 cm. The patient underwent conservative management, with closed reduction performed under general anesthesia. He was followed for five years post-injury. To our knowledge, a five-year follow-up has not been reported in the English language literature. The patient subsequently underwent a structured rehabilitation protocol, including progressive weight-bearing and activity reintegration. A five-year follow-up evaluation ruled out avascular necrosis of the femoral head. Pure traumatic anterior hip dislocations are rare injuries, typically resulting from high-energy trauma. Prompt closed reduction under general anesthesia, often guided by fluoroscopy, is the preferred management approach. If closed reduction is unsuccessful, urgent open reduction should be performed. Long-term follow-up is crucial to monitor for complications such as avascular necrosis and joint dysfunction.

## Introduction

Traumatic hip dislocations are uncommon but potentially devastating injuries in the pediatric population, accounting for approximately 5% of all hip dislocations that necessitate swift diagnosis and proper treatment [1]. Among these, anterior dislocations are significantly rarer compared to posterior dislocations, which occur nearly nine times more frequently​ [2]. The hip joint's strong ligamentous and osseous structures make dislocation an unusual event [3]. Anterior hip dislocations take place when the hip joint is forcefully shifted anteriorly, typically requiring high-energy trauma, such as motor vehicle collisions or severe falls​ [4]. However, children may sustain hip dislocations from relatively lower-energy mechanisms due to increased joint laxity and an incompletely ossified acetabulum​ [5].

Anterior hip dislocations can be further classified into superior (pubic) and inferior (obturator) types, depending on the final position of the femoral head​ [6]. The inferior-anterior (obturator) dislocation occurs when the femoral head is displaced anteroinferiorly, often into the obturator foramen, resulting in a characteristic clinical presentation of hip flexion, abduction, and external rotation​ [7]. The mechanism of injury for such dislocations typically involves forced abduction and external rotation or a direct impact on the posterior aspect of the hip​ [6].

Prompt reduction of the dislocation is crucial as delays beyond six hours increase the risk of avascular necrosis (AVN) due to compromised blood supply to the femoral head​ [4]. Management primarily involves closed reduction under general anesthesia, often performed under fluoroscopic guidance to confirm proper alignment​ [1]. If closed reduction fails, open surgical intervention is warranted to restore joint congruity ​[2]. Post-reduction, patients require close follow-up for potential complications, including AVN, chondrolysis, and joint instability​ [3]. Rehabilitation and physical therapy are essential for recovery, emphasizing the restoration of range of motion, muscle strength, and functional mobility [4].

Despite its rarity, traumatic inferior-anterior hip dislocation presents a significant clinical challenge due to its potential for long-term morbidity. A systematic approach to early recognition, urgent reduction, and structured rehabilitation is critical for optimal outcomes. This case report aims to contribute to the existing literature by discussing a unique case of inferior-anterior hip dislocation, outlining its management, and associated clinical outcomes. The patient approved written informed consent for the publication of this case. To the best of our knowledge, a five-year follow-up has not been previously documented in English-language literature.

## Case presentation

A 15-year-old adolescent male, with a normal body habitus and no previous co-morbidity, presented to the emergency department (ED) with a left hip deformity, an inability to bear weight, and significant pain. He was transported by ambulance following a direct impact to the left hip after a collision with a moving vehicle. The force of the impact resulted in hyperabduction and external rotation of the left leg. At the time of presentation, the patient was non-ambulatory and exhibited signs of a mild traumatic brain injury. The patient's vital signs were stable, and the head, neck, chest, abdomen, and spine examination revealed no abnormalities. There was no prior history of joint dislocations or laxity.

**Figure 1:**
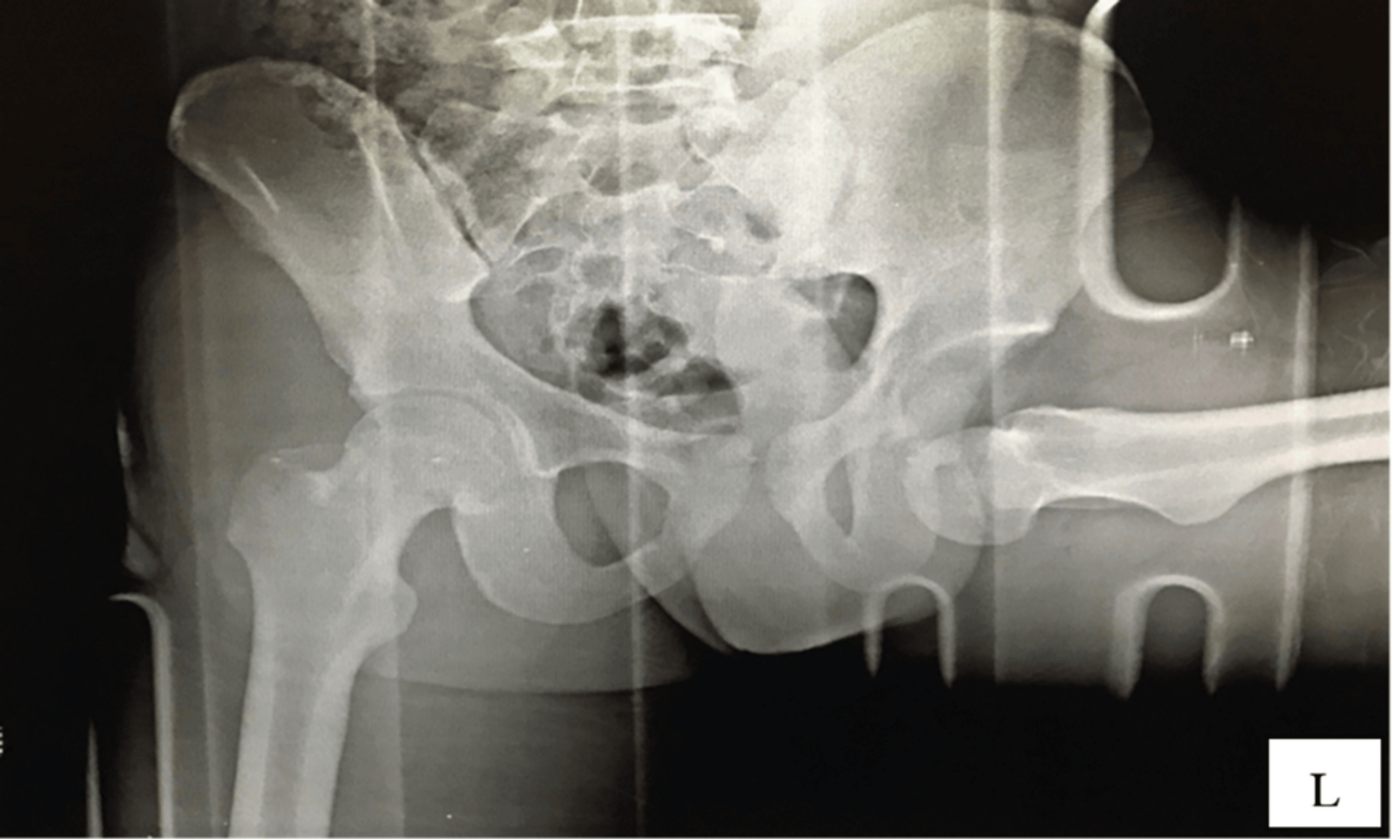
Preoperative anteroposterior radiography of the pelvis with both hips reveals an inferior-anterior dislocation of the left femur.

On clinical examination, the left lower limb was positioned in hyperflexion, 90° of abduction, and external rotation at the hip. The neurovascular assessment was normal, showing strong distal pulses. A comprehensive motor assessment was limited due to the child's discomfort and limb deformity; however, spontaneous dorsiflexion and plantarflexion of the left ankle were observed. Radiographic imaging confirmed a closed hip dislocation without associated fractures (Figure 1). The femoral head was displaced inferior to the acetabulum, situated within the obturator foramen. No fractures or physeal separations were identified.

**Figure 2:**
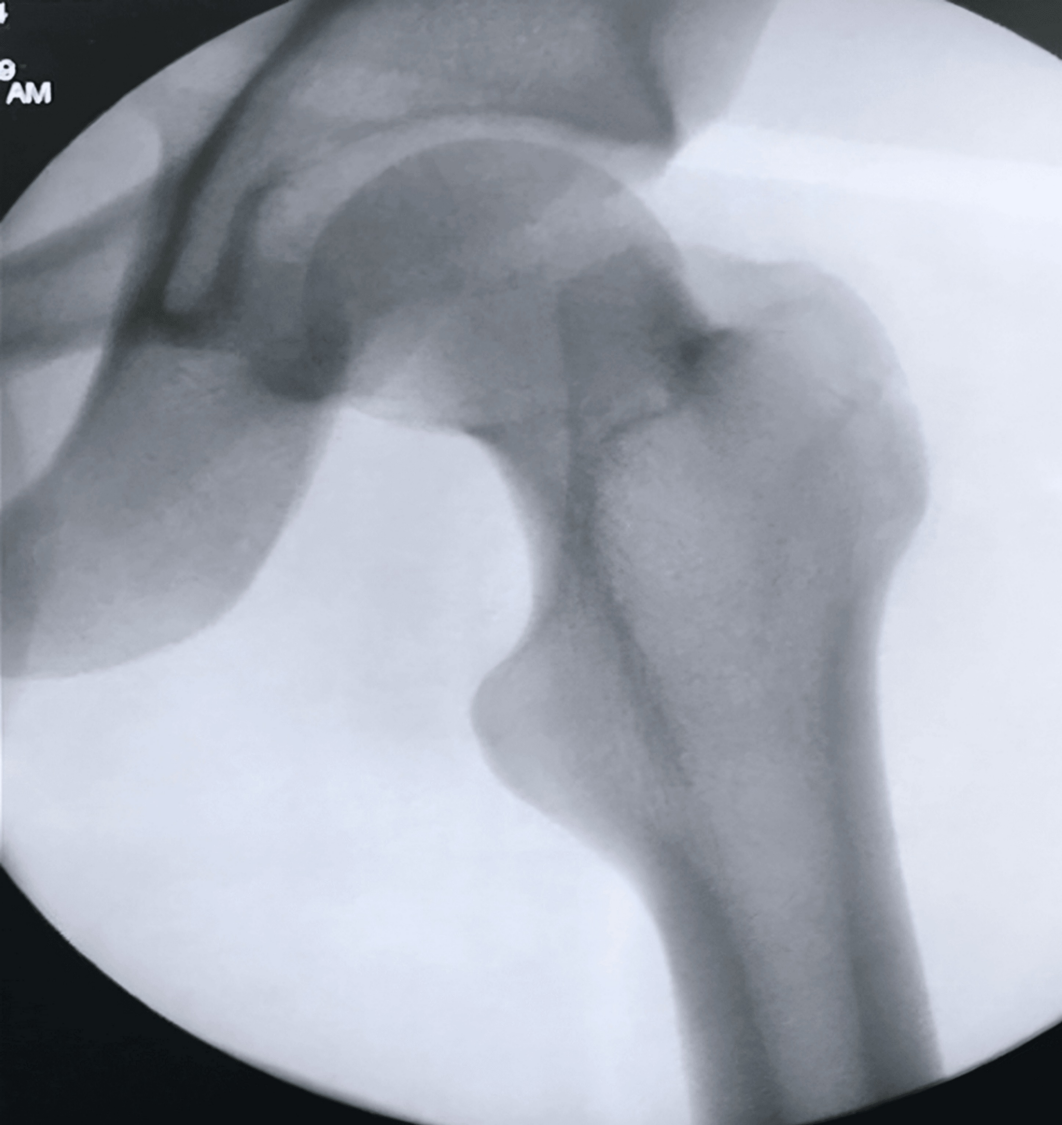
Perioperative anteroposterior fluoroscopy after close reduction shows relocation.

The patient underwent an urgent closed reduction under general anesthesia, performed two hours post injury. In the operating room, he was positioned supine, and the knee was flexed to relax the hamstring muscles. Distal traction was applied to the femur, followed by gentle adduction and internal rotation, while an assistant exerted direct pressure on the femoral head to achieve reduction. Intraoperative fluoroscopy confirmed a successful reduction, and the patient was placed on skin traction with a 2 kg weight (Figure 2).

A post-reduction assessment confirmed that the limb remained neurologically intact, demonstrating preserved motor function, sensation, and strong distal pulses. The patient was monitored overnight for serial neurovascular evaluations.

**Figure 3:**
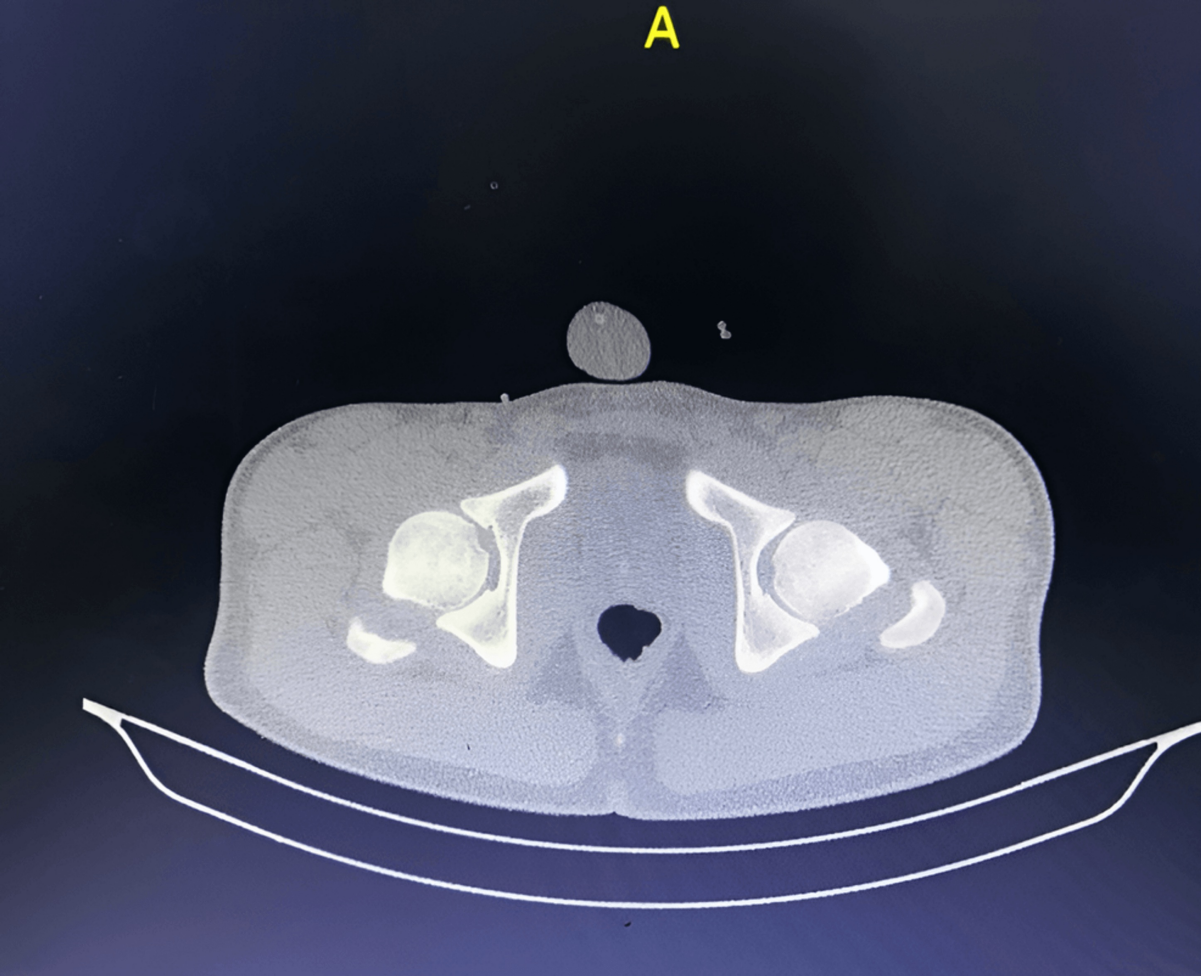
Axial computed tomography views after reduction showed relocation of the femoral head without fracture.

**Figure 4:**
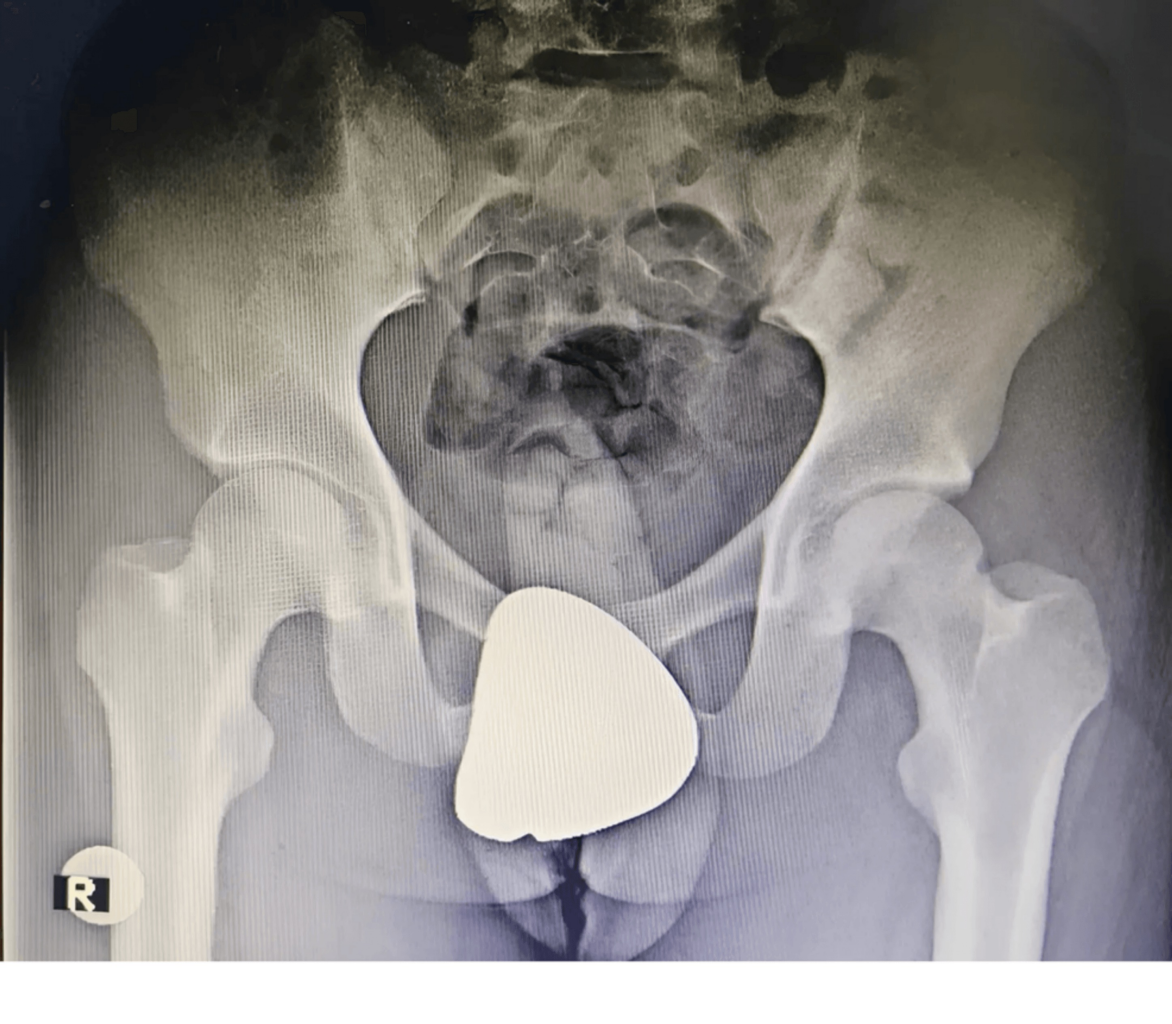
Anteroposterior radiograph next day after close reduction shows relocation of the femoral head.

Post-reduction radiographs and computed tomography (CT) performed the following day confirmed a concentric reduction of the hip, with no evidence of associated fractures or intra-articular fragments (Figures 3-4). Additionally, there was no evidence of physeal displacement. The patient was discharged home seven days post-injury after a comprehensive evaluation and monitoring period. At the time of discharge, the 2 kg skin traction was discontinued.

Following discharge, the patient adhered to a structured rehabilitation protocol, which included a gradual weight-bearing regimen and progressive functional reintegration.

Phase 1: Acute Phase (Zero to Two Weeks Post Injury)

Weight-bearing status: Non-weight-bearing with crutches. Immobilization: skin traction for pain management the first three days; pain control: nonsteroidal anti-inflammatory drugs (NSAIDs) and cryotherapy; range-of-motion (ROM) exercises: passive ROM initiated within a pain-free range; and isometric exercises: quadriceps and gluteal sets to prevent muscle atrophy.

Phase 2: Early Rehabilitation (Two to Six Weeks Post Injury)

Weight-bearing progression: Partial weight-bearing as tolerated. ROM progression: a gradual increase in hip flexion, abduction, and internal rotation; strength training: initiation of closed-chain exercises (e.g., seated leg presses, mini squats); and neuromuscular re-education: balance and proprioception exercises.

Phase 3: Strengthening and Functional Recovery (Six to 12 Weeks Post Injury)

Full weight-bearing restoration: Active ROM exercises with resistance bands. Progressive strengthening: leg presses, lunges, and hip abductor/adductor exercises; and gait training: focus on normalizing walking patterns without compensatory mechanisms.

Phase 4: Return to Sports (Three to Six Months Post Injury)

Advanced strength training: Plyometric drills and sport-specific movements. Running progression: jogging, followed by sprinting drills; agility and endurance training: cutting, pivoting, and multidirectional movement drills; and clearance for competition: based on functional assessment, pain resolution, and imaging clearance.

Weight-bearing was initially restricted with the use of crutches, and hip abduction was limited for one month. At the two-week follow-up, the patient reported minimal hip pain at rest and while walking. The ROM assessment revealed 90° of flexion and normal extension, with mild discomfort during passive abduction. Neurovascular status remained intact.

By the one-month follow-up, the patient was pain-free, demonstrating excellent hip mobility with no discomfort during passive abduction. He remained neurovascularly intact and reported no functional deficits in the left hip. At the three-month follow-up, the patient had fully regained weight-bearing capacity and had resumed light physical activities, including jogging. At the six-month follow-up, he had progressed to playing basketball and participating in all functional activities without pain or movement restrictions. Radiographic evaluation showed no evidence of AVN.

**Figure 5:**
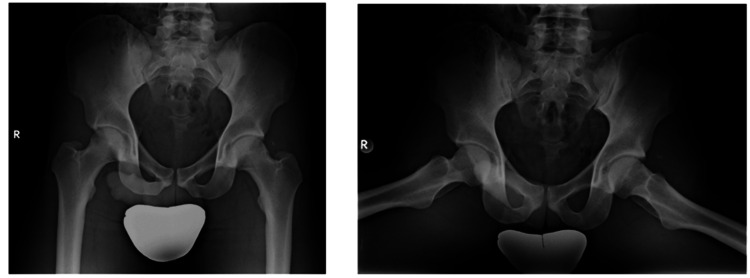
Final follow-up radiographs at five years showing no avascular necrosis and joint dysfunction.

Over a five-year follow-up period, serial imaging assessments were conducted to monitor for AVN of the femoral head (Figure 5). At the five-year mark, the patient remained pain-free, exhibited no functional limitations, and had fully resumed athletic participation without hip instability or discomfort.

## Discussion

From an anatomical point of view, the hip joint is a ball-and-socket articulation, with the femoral head only partially covered by the acetabulum [3]. Due to the depth of the acetabulum, which is further reinforced by the labrum, as well as the thick joint capsule and robust muscular support, the hip is one of the most stable joints in the body and is less prone to dislocation [3]. Ligamentous stability is provided by strong capsular ligaments, extending from the acetabulum to the femoral neck and intertrochanteric region. Children are more prone to joint dislocation as less force is required due to their soft, flexible acetabulum and increased ligamentous laxity [8]. The iliofemoral ligament reinforces the joint anteriorly, while the ischiofemoral ligament provides posterior support and the short external rotator muscles, which adhere to the posterior capsule, contribute further to the joint’s overall stability [3].

Congenital hip dislocation in pediatric patients is frequently observed either as an isolated condition or in association with other congenital disorders, including Down syndrome, Ehlers-Danlos syndrome, Weaver syndrome, and Prader-Willi syndrome [9-11].

A considerable amount of force is needed to compromise the strong ligamentous capsule that stabilizes the hip joint, which is among the most stable joints in the body [8]. In children, the force needed to cause a hip dislocation can vary from minor incidents, such as tripping or falling from a standing height to more severe high-impact injuries such as those sustained in motor vehicle accidents [4,5,12,13]. Particularly in young children, hip dislocation can occur with significantly lower energy trauma, under five years old, as the acetabulum at this stage consists largely of soft, pliable cartilage, and ligamentous laxity is more pronounced, making the joint more susceptible to displacement [3]. As children grow older, greater force will be required for hip dislocation (higher-force trauma); this is due to a decrease in the cartilage-to-bone ratio and the increasing rigidity of periacetabular structures [8]. The acetabulum becomes more ossified and less flexible, while the ligaments also gain stiffness, enhancing joint stability [8]. Consequently, older children typically require more significant trauma for a hip dislocation to occur [8].

The hip can dislocate in different directions, with posterior dislocation being the most common, followed by central and anterior dislocations. As previously mentioned, traumatic pediatric hip dislocations comprise approximately 5% of all traumatic hip dislocations [1]. In the pediatric population, anterior hip dislocations are even less common [1]. Possible complications include AVN in approximately 10% of cases [12], associated fractures occurring in up to 40% of patients [7], and neurological or vascular impairment in nearly 25% of cases [12]. Additionally, articular cartilage damage is observed in around 6% of cases, while intra-articular fragments trapped within the acetabulum are reported in up to 25% of patients [12,13].

Given the potential severity of these complications, hip dislocations require urgent medical attention, with reduction recommended within six hours post injury to minimize the risk of AVN [12]. Delayed reduction significantly increases the likelihood of AVN, a complication that poses greater challenges in pediatric patients due to the complexity of treatment options [7]. To reduce the risk of AVN, prompt reduction should be performed as soon as the patient receives adequate sedation [12]. Studies indicate that the incidence of AVN is 4.8% in patients who undergo hip reduction within six hours, whereas the risk dramatically rises to 52.9% when reduction is performed after six hours [14].

Ahmad et al. described an eight-year-old girl who sustained an obturator-type hip dislocation following a minor fall while playing [1]. Closed reduction was promptly performed under general anesthesia within six hours of injury, followed by immobilization in skin traction for three weeks [1]. The patient demonstrated a full, pain-free ROM at follow-ups conducted at three, six, and 12 months [1]. No signs of AVN or other complications were observed, emphasizing the importance of early intervention for optimal recovery [1].

Gupta et al. underscored the importance of early diagnosis, prompt reduction, and structured rehabilitation in managing pediatric hip dislocations effectively [3]. In their case report, a 12-year-old boy sustained a traumatic hip dislocation following a hyperabduction injury when his left foot became entangled in a staircase while catching kites [3]. The radiographic evaluation confirmed inferior-anterior hip dislocation without fractures, and the hip was successfully reduced under intravenous sedation within two hours post injury [3]. Skin traction was maintained for two weeks, followed by a gradual rehabilitation program [3]. At a two-year follow-up, he remained asymptomatic, with normal radiographic findings and no evidence of AVN, osteoarthritis, or structural abnormalities [3].

Avery et al. in their case underscored the importance of early recognition, timely intervention, and long-term follow-up in pediatric hip dislocation management [7]. A nine-year-old boy sustained a left hip dislocation after an awkward fall while running, resulting in pain, deformity, and an inability to bear weight [7]. A closed reduction was performed under sedation within three hours post injury, and the patient followed a structured rehabilitation plan, including restricted movement and gradual weight-bearing over four weeks [7]. Follow-up assessments at one, three, and six months, as well as two-year imaging studies, showed complete recovery, no AVN, and a return to full physical activity [7].

Khongwir et al. presented a case of a four-year-old boy who sustained an anterior hip dislocation following a traumatic injury, along with associated polytrauma, including a flail chest and pneumothorax [8]. Early closed reduction under general anesthesia was performed within four hours of admission, followed by immobilization in a hip spica and subsequent skin traction [8]. The patient demonstrated full, pain-free mobility at follow-ups after three and six months, with no signs of AVN or other complications [8]. Additionally, this case highlights the importance of early diagnosis using advanced imaging and timely intervention to ensure optimal recovery in pediatric hip dislocations [8].

This case report details a 15-year-old male who sustained an obturator-type hip dislocation following a direct impact to the left hip in a vehicular collision. The patient presented with severe pain, hip deformity, and an inability to bear weight, though neurovascular assessment remained intact. Radiographic imaging confirmed a closed dislocation without associated fractures. An urgent closed reduction under general anesthesia was performed within two hours of injury, followed by skin traction and a structured rehabilitation protocol.

The patient demonstrated progressive recovery through a staged rehabilitation approach, transitioning from non-weight-bearing to full functional reintegration. By the six-month follow-up, he had resumed sports activities without pain or mobility restrictions. Radiographs after five years showed no evidence of AVN or joint instability, confirming a successful long-term outcome. This case highlights the importance of early diagnosis, prompt reduction, structured rehabilitation, and long-term monitoring to optimize recovery in pediatric hip dislocations. To the best of our knowledge, a five-year follow-up has not been previously documented in English-language literature (Table 1).

**Table 1:** Review of the literature.

Study	Year	Age	M/F	Mechanism of injury	Associated injuries	Treatment	Follow-up	Outcome
Ahmad et al. [1]	2015	8 years	Female	Trivial fall while playing	None	Closed reduction under GA (general anesthesia), skin traction for 3 weeks	12 months	Full recovery, no AVN (avascular necrosis), full ROM (range of motion), MRI confirmed no complications
Gupta et al. [3]	2013	12 years	Male	Fell while catching kites, left foot entangled in staircase causing hyperabduction injury	Minor abrasions on the left foot	Closed reduction under intravenous sedation within 2 hours	2 years	Full recovery, no pain, no movement restriction, normal radiographs
Avery et al.(7)	2013	9 years	Male	Fell awkwardly while running, forcing the left leg into hyperabduction and external rotation	Mild strain left quadratus femoris and focal bone contusion right posterior superior iliac spine (without fracture)	Closed reduction under sedation	2 years	Full recovery, no pain, normal hip function, no signs of AVN
Khongwir et al. [8]	2018	4 years	Male	Trauma (polytrauma, likely high-energy impact)	Left pneumothorax (flail chest)	Closed reduction under GA, hip spica, then skin traction	6 months	Painless full range of motion, ambulating unassisted, no AVN
Present case	2025	15 years	male	Direct impact from vehicle collision	Mild traumatic brain injury	Closed reduction under GA, skin traction	5 years	Full recovery, no AVN, no functional limitations, return to sports

## Conclusions

Traumatic anterior hip dislocations are rare injuries, typically resulting from high-energy trauma. This case report and review of the literature underscores the importance of early diagnosis, timely intervention, and long-term monitoring in the management of pediatric hip dislocations. Prompt closed reduction under general anesthesia, often guided by fluoroscopy, is the preferred management approach. Reduction is recommended within six hours post injury to minimize the risk of AVN. If closed reduction is unsuccessful, urgent open reduction should be performed. Long-term follow-up is crucial to monitor for complications, such as AVN and joint dysfunction.
